# 627. Epidemiology and Outcomes of Pavement Burns Among Unhoused Patients

**DOI:** 10.1093/jbcr/irag033.453

**Published:** 2026-04-06

**Authors:** Samuel A Matthys, Alexander Rowan, Ryan Grinnell, Vidhani Goel, Brenna Chen, Lateef Omidiji Jr., Avni Joshi, Shane Jung, Stephanie Martinez, Kavita Batra, Rabia Nizamani

**Affiliations:** Leon S. Peters Burn Center, Las Vegas, NV; Leon S. Peters Burn Center, Las Vegas, NV; Kirk Kerkorian School of Medicine at University of Nevada, Las Vegas, NV; Kirk Kerkorian School of Medicine at University of Nevada, Las Vegas, NV; Leon S. Peters Burn Center, Las Vegas, NV; Kirk Kerkorian School of Medicine at University of Nevada, Las Vegas, NV; Leon S. Peters Burn Center, Las Vegas, NV; Leon S. Peters Burn Center, Las Vegas, NV; University of Nevada, Las Vegas, NV; Kirk Kerkorian School of Medicine at University of Nevada, Las Vegas, NV; UMC Lions Burn Care Center, Las Vegas, NV

## Abstract

**Introduction:**

Unhoused individuals are disproportionately vulnerable to climate-related injuries due to prolonged outdoor exposure and limited access to shelter. In desert climates, summer temperatures often exceed 110°F, with pavement surface temperature exceeding 150°F. Even brief contact can result in significant partial and full thickness burns. At our regional burn center, although the unhoused comprise only 0.33% of the local population, they disproportionally account for 13% of pavement burn admissions. This study examines the epidemiology, hospital course, and outcomes of pavement burn injuries among unhoused patients, highlighting a preventable and growing public health concern.

**Methods:**

A retrospective review of burn registry data from 2014–2024 identified unhoused patients with pavement burn injuries. Demographics, burn size (TBSA%), length of stay (LOS), ICU admission, mechanical ventilation, and mortality were recorded. Continuous variables were compared with t-tests and categorical variables with chi-square or Fisher’s exact tests. Trends over time were assessed using chi-square tests for trend, with significance at *p*<.05.

**Results:**

Among 95 unhoused contact burn patients, 92 (96.8%) sustained pavement burns. Mean age was 49.9 ± 12.9 years, 87.4% were male, and most injuries occurred in summer (85.3%), peaking in 2024 (38.4%, *p*<.001). (Table 1) Mean TBSA was 5.4 ± 4.5%, GCS 12.9 ± 4.1, and initial body temperature higher than those who are housed (37.5 vs. 37.0°C, *p*=.004). Hospital LOS was longer in unhoused patients (25.8 vs. 15.3 days, *p*=.032). ICU admission (29.5%) and ventilator use (18.9%) were more frequent but not significant. Mortality was 3.2%, similar to housed patients. (Table 2).

**Conclusions:**

Pavement burns are a growing source of morbidity in the unhoused community. While TBSA and ICU use were higher but not statistically different, hospital stay was significantly longer reflecting challenges with disposition, housing insecurity, and complex wound care. The sharp rise in admissions in recent years highlights the urgent need for targeted prevention and heat-response strategies for unsheltered populations.

**Applicability of Research to Practice:**

Pavement burns in the unhoused community are a growing public health concern that will likely require injury prevention through further governmental and community outreach, especially during summer months. Further study in other desert climate regions should be done to compare incidence and outcomes to determine the scope of this issue, and to determine best practices for injury prevention.

**Funding for the study:**

N/A.

